# Study of Self-medication among First and Seventh Semester Medical and Dental Undergraduate Students of Tertiary Care Teaching Hospital in Nepal: A Descriptive Cross-sectional Study

**DOI:** 10.31729/jnma.5385

**Published:** 2021-01-31

**Authors:** Jyoti Tara Manandhar Shrestha, Dilip Kumar Kushwaha, Saurabh Tiwari

**Affiliations:** 1 Department of Pharmacology, Kathmandu University School of Medical Sciences, Dhulikhel, Kavre, Nepal; 2 Kathmandu University School of Medical Sciences, Dhulikhel, Kavre, Nepal

**Keywords:** *dental*, *medical students*, *practice*, *prevalence*, *self medication*

## Abstract

**Introduction:**

Although appropriate self-medication can ease minor illness and is time and cost-effective, it can lead to irrational drug use and increased resistance, leading to prolonged morbidity. Inclined towards medical information and drug indices, medical students have an open arena for self-medication practice. This study was conducted to find the prevalence of self-medication among first and seventh semester medical and dental students in a tertiary care hospital.

**Methods:**

A descriptive cross-sectional study was conducted among medical and dental undergraduates from July 2020 to August 2020 after receiving ethical clearance from the Institutional Review Committee of Kathmandu University School of Medical Sciences (IRC Approval Number: 35/20). A questionnaire was responded to by participants through a google form. Participants were enrolled through the convenience sampling method. Data were collected and entered in Microsoft Excel and analyzed using Statistical Package for Social Sciences version 25.

**Results:**

Out of 199 respondents, the prevalence of self-medication was 100 (50.3%) (46.76-53.84 at 95% Confidence Interval). First semester medical 36 (73.5%) and dental undergraduates 24 (80%) had higher practice. Seventh-semester medical students 14 (51.9%) usually self-medicated within one day of onset of symptoms. Headache 47 (47%) was the most common indication. Analgesics 62 (62%) were most commonly used drugs procured most commonly from pharmacies 114 (57.3%). Dosage form was drug selection criteria for 120 (60.3%) students.

**Conclusions:**

Since self-medication is crammed with serious health hazards, proper exposure to the topic should be given to medical, dental students, and pharmacists. The implication of self-medication into the pharmacology syllabus is a must.

## INTRODUCTION

Self-medication is the treatment of self-recognized disorders or symptoms using drugs or continuation of medicines prescribed for recurring physical or psychological ailments outside the formal health care system without a doctor's prescription.^1–3^ Appropriate self-medication can ease minor illness and is time and cost-effective within limited resources.^4^ Spurious self-medication can lead to irrational drug use, increased pathogen resistance, health hazards, adverse drug reaction, and prolonged morbidity.^5^

Medical students have better access to health care information and facilities, creating an open arena for self-medication practice.^6^ Inclined towards medical information and drug indices, they get a ground to perform self-diagnosis and self-medication. Studying self-medication practice among medical students is crucial as they are future drug prescribers and health educationalists and self-medication practices that may cause health hazards and morbidity, thus degrading overall health quality.^7^

This study was conducted to find the prevalence of self-medication among first and seventh semester medical and dental students in a tertiary care center.

## METHODS

A descriptive cross-sectional study was conducted in medical and dental students of Kathmandu University School of Medical Sciences, Dhulikhel during July 2020 to August 2020 after receiving approval from the Institutional Review Committee (IRC) of the same institution (IRC Approval Number: 35/20 Nepalese students of age greater than or equal to 17 years studying in the first and seventh semester medical and dental undergraduate course were included in the research; foreigners being excluded. Students not willing to participate in the research and students on chronic medication were also excluded. The sample size was calculated as:

$$\begin{array}{ll} \text{n} & ={\text{Z}}^{\text{2}}\text{p(1}-\text{p)}/{\text{e}}^{\text{2}}\\ & ={\left(1.96\right)}^{2}\times 0.76\times (1-0.76)/{\left(0.05\right)}^{\text{2}}\\ & =280.3\\ & \approx 280 \end{array}$$

Where,

n = sample size
Z = 1.96 at 95% Confidence Interval (CI)
p = prevalence from a previous study (76.6%)^8^
e = margin of error, 5%

Since the study was done in a finite population of 228. Sample size was adjusted as,

n = n1/ [1+ ( n1-1)/N]

The corrected sample size of the source population of 228 students was = 280/ [1 +(280-1)/228]

= 125.9

≈ 126.

After adding a 20% non-response rate, the final sample size calculated was 151. However, the study was conducted among 199 students, and data was collected using the convenience sampling method.

Since the research was conducted during the COVID-19 pandemic, participants were asked to join a Zoom meeting conducted by the investigators for research keeping the value of social distancing in mind. They were told about the research and the time duration required to complete it. After explaining about the research, they were asked for consent, which was prepared by google form.

After receiving the consent, they were given the link to the questionnaire.^9^ It was assured that questions ran out of confusion, and they were requested to fill the form afterward. The questionnaire contained questions on the respondents’ demographic information such as age, gender, semester, and Socioeconomic variables such as health-seeking behavior names and sources of drugs used for self-medication, type of illness, and factors influencing self-medication practices.

The data collected through google form were recorded in Microsoft excel and was analyzed using Statistical Package for the Social Sciences (SPSS) version 25, and descriptive parameters were analyzed and depicted in numbers, percentages, and graphs.

## RESULTS

**Figure 1:**
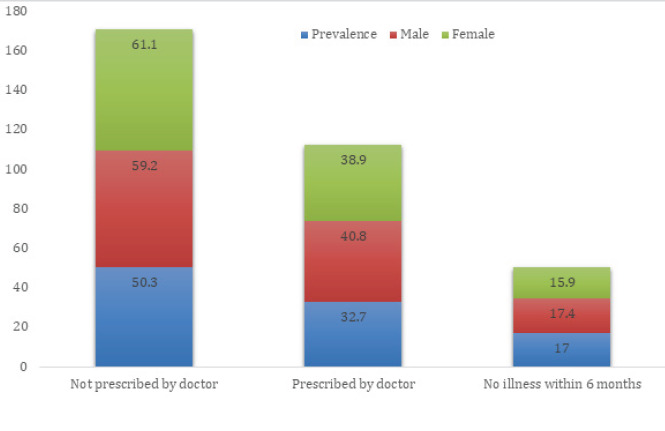
Prevalence of self-medication among different gender.

Out of 199 medical and dental students, the prevalence of the practice of self-medication within 6 months was 100 (50.3%) (46.76-53.84 at 95% CI). Fifty-eight (61%) participants were females. The mean age of students was 20.82±1.75 years. Out of 199 students, 86 (43.2%) were male, and 113 (56.8%) were female. (Figure 1).

Overall, the first semester, i.e., 36 (73.5%) medical and 24 (80%) dental students were found self-medicating, which was quite higher than 28 (46.7%) medical and 12 (44.4%) dental students of the seventh semester. Interestingly, 14 (51.9%) medical and 5 (41.7%) dental students from the seventh semester were found to be practicing self-medication early within 1 day of onset of symptoms. Moreover, 171 (86%) took medication within 7 days.

**Figure 2:**
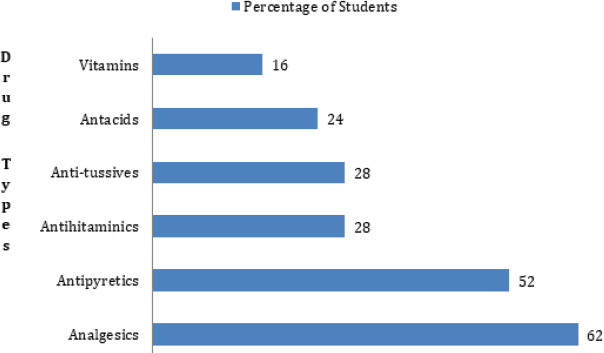
Types of self-medicated Drugs.

Analgesics 62 (62%) were the most commonly self-medicated drug (Figure 2).

**Figure 3:**
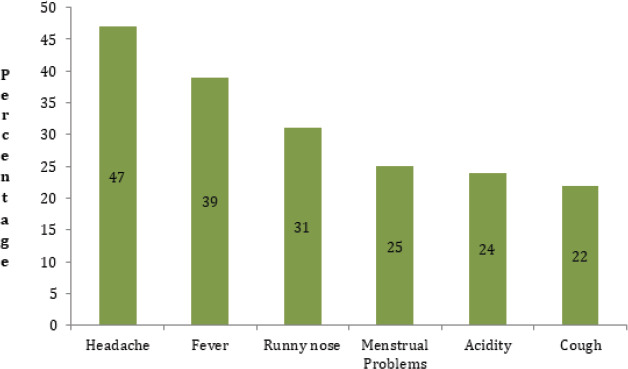
Common Indications of Self-medication.

The most common indications for self-medication was found to be headache 47 (47%) (Figure 3).

The main source of procurement was Pharmacies and Pharmacist's advice 114 (57.3%) followed by Pharmacology learning 89 (44.7%), expertise from previous illness 70 (35.2%), family and friends 60 (30.2%), and leftover medicines 45 (22.6%).

The main reason for self-medication, as stated by students, were minor illness 64 (64%), previous exposure from disease 39 (39%), instant relief 33 (33%), and knowledge from pharmacology teaching 28 (28%).

**Figure 4:**
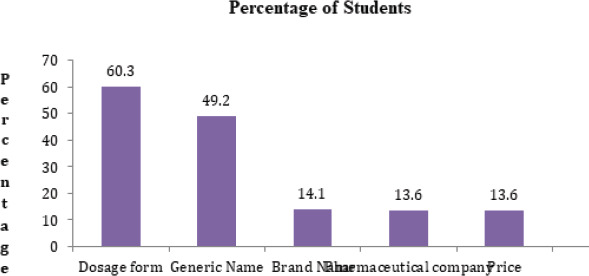
Factors considered during self-medication.

Most of the students preferred dosage form 120 (60.3%) during selection. Generic name 98 (49.2%) and brand name 28 (14.1%) were also considered (Figure 4). First semester students 20 (26.35%) preferred brand name to generic name, which was preferred only by 3 (4.55%) of seventh-semester students.

Out of total students, 47 (23.6%) considered self-medication as safe, 33 (16.6%) encouraged self-medication, and 191 (96%) felt the urge of implications of self-medication and its consequences in the syllabus of pharmacology. Fortunately, 124 (63.6%) did not prescribe medicine to others. Almost all 191 (91%) reported the fact that self-medication leads to irrational use of drugs.

Among the students who considered self-medication unsafe, 124 (81%) described self-medication as a factor for drug resistance, 120 (78.4%) as an adverse drug reaction, and 95 (62.1%) as masking of an underlying disease.

Almost all 194 (97.5%) students agreed that self-medication of antibiotics leads to the emergence of resistance, and 162 (81%) considered early relief as the main reason for the incomplete course of antibiotics. However, 60 (30.2%) students were unaware of the complete course of antibiotics.

It was not uncommon to find that 53 (70.9%) of the first semester were unaware of the adverse effects of the drug they used, but the fact that 25 (32.85%) of the seventh semester were unaware was astonishing. Besides, 31 (41.2%) of the first semester and 12 (16.15%) of the seventh semester were unaware of the correct dosage of self-medicated drugs.

Regarding over-the-counter drugs, 24 (32.75%) of first and 6 (8.25%) of the seventh semester were unaware.

## DISCUSSION

The study focused on medical and dental undergraduates from the first and seventh semester who were not eligible to prescribe medicine legally. The overall prevalence for the custom of self-medication in those having illness within 6 months was 50.3% (100 out of 199 students), which is in accordance with Nepalese study conducted in Eastern Nepal 51.1%^10^ but higher than the study done in Western region 38.2%^11^ and lower than Mid-western region 61.5%,^12^ Central region 76.6%, 8 83.3%,^13^ and 84%^14^. The study is also congruent with studies conducted in different parts of India like 40.9%^15^ in Pune, 57.05%^5^ in West Bengal, 65%^16^ in Kolkata, 82.3%^17^ in Ahmedabad, 83%^18^ in North India, 84%^19^ in South India, and other countries like 59%^20^ in Syria, 62.9%^21^ in Egypt, 79.9%^22^ in Serbia.

The variation of the prevalence of self-medication may be due to various factors like differences in the locality of the study population (medical and dental students), research methods, definitions used, sampling techniques, availability of the drugs in the local pharmacy without valid prescriptions, regulation of policy regarding the availability of over the counter (OTC) drugs, acquired knowledge of medicine, convenience time-saving nature, and cultural differences thus, true comparison is not possible.

Self-medication practice was generally expected to increase from first to final year due to the development of confidence regarding knowledge about drugs, diseases, prognosis, and prescription patterns, as evidenced by various studies that were previously conducted.^5,10,15,21^ Also, equal prevalence between first to final semester have been seen.^23,24^ But in contrast to these findings, our study concluded that First semester students self-medicated more than seventh semester^8,13,17,25,26^ and most of the seventh-semester students self-medicated within one day of onset of symptoms. This may be attributed to an increase in awareness and knowledge about deleterious health hazards due to self-medication practice with an increase in years of education. First semester students have lesser knowledge about drugs and self-medication compared to seventh semester making them more vulnerable.^8^ Seventh semester students are more confident to diagnose the diseased condition, prognosis, and treatment after being taught in clinical classes. This explains the basis of self-medication within one day of illness in seventh-semester students.

Females were found to be self-medicating slightly more than males, which is similar to other studies.^8,13,22^ This might be due to females’ perception of medication and their hesitancy to consult the doctor for their illness.^8^ But it is in contrast with studies which revealed male self-medicated more.^10,12,20,27^

The most common indications for self-medication were headache (47%) which is similar to a study conducted in various regions^8,20,24^ but has been opposed by research where common cold were the major indications.^10,14,15,27^ The variation in climatic conditions may have resulted in the difference.

Analgesics (62%) followed by antipyretics (50%) were self-medicated routinely. Our study's Congruency was maintained by studies where analgesics and antipyretics were commonly self-medicated^8,13,23,29^ but this contrasted to other studies where antibiotics were commonly^5,10,28,30^ sedatives were commonly prescribed^31^ and complementary and alternative medicines were also prescribed.^32^ Congruity may be due to similar indications for self-medication and similar policy regarding the availability of similar drugs without prescription, while the difference in policy and knowledge regarding self-medication may have created the contrast. More than half (60.3%) of the students preferred dosage form to generic (49.2%) and brand names (14.1%); oral route being more common, which is in agreement with Indian study.^25^

Preference of generic name 49.2% followed brand name 14.1% which was also seen in other studies.^33^ Medical students are usually taught about drug therapy for particular illnesses by their generic names and not by brand names. This may have caused the generic name to be popular among medical students. But the discrepancy was maintained by a study where brand name 82% followed generic name 18%.^15^

The major reason for self-medication was minor illness (64%) and previous medication experiences due to similar illnesses (39%). This was a similar finding revealed from other studies.^13,18^ Time saving,^8,17^ Confidence of gaining sufficient knowledge,^31^ lack of knowledge about the disadvantages of self-medication^11^ were other important findings presented in other studies.

Pharmacy (57.3%) was the major source of procurement, and this is in accordance with other studies.^8,10,25,26^ Pharmacist's advice (100%) was the major source of procurement of antibiotics for medical and dental undergraduates of the first semester, but none of the seventh-semester students relied on Pharmacist's advice for self-medication. However, some students also procured from pharmacology knowledge (44.7%), exposure from previous illness (35.2%), family, relatives, and friends (30.2%). This signifies the importance of proper knowledge of pharmacology for pharmacists to dispense drugs accurately. Some studies also showed the internet 44.2% as a rising source of procurement.^34^ This demands the need for authentic websites on which people can rely to know about the drugs.

In our study, 16.6% of the students encouraged, and 23.6% considered self-medication safe, similar to other studies.^35^ This may be due to proper knowledge acquired regarding deleterious effects of self-medication during medical lectures. Opposing this view, there were some studies where more than half of respondents encouraged self-medication rather than discouraging it.^8,17,21^ Although medical and dental students are allowed to prescribe medicines only after passing their licensing examinations which are taken after completion of their undergraduate courses, 36.4% prescribed drugs to others, which was a harmful practice.

Regarding the complete antibiotics course, more than half, 69.8% were aware which was supported by previous studies.^36^ Some studies revealed as low as 31%18 and as high as 91.7%^25^ awareness among respondents. Most of the students 81%, considered early relief to be the major reason for the incomplete course of antibiotics. This was also evidenced in Egyptian Study.^21^

Drug resistance (81%) was found to be the most common cause for unsafe self-medication; others being adverse drug reaction (78.4%) and masking of underlying diseases (62.1%), which were similar to other studies.^8^ Majority of the seventh semester, including 80% medical and 54% dental students, were aware of the adverse effects of medications they consumed, which satisfies other studies’ findings.^25^ However, 36.2% of medical and 22% of dental students were unaware in our study. This warrants the dire need for the implementation of self-medication and its consequences into the Pharmacology syllabus. Almost all (97.5%) of students agreed that antibiotics’ self-medication leads to the emergence of resistance. Almost all students admitted to the fact that custom of self-medication leads to irrational use of drugs and felt the urge of the implication of self-medication and its consequences in Pharmacology syllabus at undergraduate level.^8^

Generalization of findings from this study may not be credited, and a causal relationship cannot be established as it was conducted in a single institute within a small population. Only medical and dental students were included without nursing, physiotherapy, and other paramedical faculties. Since the survey was online, there might have been a misinterpretation of questions and missing of data. Recall bias cannot be completely avoided. Respondents’ willful falsification may be another limitation accompanied by mutual influence, although students were encouraged to complete it individually. Psychometric evaluation of students was not done. Considering all these limitations, a multicentric study should be conducted to overcome the dearth of data and reveal various factors influencing self-medication seem to be of dire need and regulation of strict policies to prohibit supply of medicines without valid prescriptions.

## CONCLUSIONS

The study revealed the urge of the implication of self-medication and its consequences into the Pharmacology syllabus as most of the medical and dental students from the first semester were found to have been adequately practicing it. Analgesics were the most commonly self-medicated drug; Headache being the most common indication. Most of the seventh-semester students self-medicated within one day of illness. The pharmacy was the major source of procurement. Since self-medication is crammed with serious health hazards, proper exposure to the topic should be given to medical, dental students, and pharmacists, and the burden of the problem should be assessed by large and diverse studies in various health care settings. Strict regulation and policy should be made for the sale of over the counter (OTC) drugs.
